# Technical Assistance Received by Older Adults to Use Commercially Available Physical Activity Monitors (Ready Steady 3.0 Trial): Ad-Hoc Descriptive Longitudinal Study

**DOI:** 10.2196/47891

**Published:** 2023-11-22

**Authors:** Elizabeth A Choma, Shannon Hayes, Beth A Lewis, Alexander J Rothman, Jean F Wyman, Weihua Guan, Siobhan K McMahon

**Affiliations:** 1 Department of Physical Therapy, Whitworth University, Spokane, WA, United States; 2 School of Nursing, University of Minnesota, Minneapolis, MN, United States; 3 School of Kinesiology, University of Minnesota, Minneapolis, MN, United States; 4 Department of Psychology, University of Minnesota, Minneapolis, MN, United States; 5 Division of Biostatistics, School of Public Health, University of Minnesota, Minneapolis, MN, United States

**Keywords:** wearable device, digital health, physical activity monitor, PAM, older adult, intervention, physical activity, usability, technical assistance, supportive structures, monitoring, promote, community, support

## Abstract

**Background:**

Despite evidence that regular physical activity (PA) among older adults confers numerous health and functional benefits, PA participation rates are low. Using commercially available wearable PA monitors (PAMs) is one way to augment PA promotion efforts. However, while expert recommendations exist for the specific information needed at the beginning of PAM ownership and the general ongoing need for structures that support as-needed technical troubleshooting, information is lacking about the type, frequency, and modes of assistance needed during initial and long-term ownership.

**Objective:**

This paper describes problems reported and technical assistance received by older adults who used PAMs during the 18 months they participated in a community-based PA trial: Ready Steady 3.0 (RS3).

**Methods:**

This was an ad-hoc longitudinal analysis of process variables representing technical problems reported and assistance received by 113 RS3 study participants in the 18 months after their orientation to PAMs. Variables included date of contact, problem(s) reported, mode of technical assistance, and whether the equipment was replaced. The descriptive analysis included frequencies and incidence rates of distinct contacts, types of problems, and technical assistance modes.

**Results:**

On average, participants were aged 77 (SD 5.2) years. Most identified as female (n=87, 77%), reported experience using smartphones (n=92, 81.4%), and used the PAM between 2 and 18 months. Eighty-two participants (72.6%) reported between 1 to 9 problems with using PAMs, resulting in a total of 150 technical assistance contacts with a mean of 1.3 (SD 1.3) contacts. The incidence rate of new, distinct contacts for technical assistance was 99 per 100 persons per year from 2018 to 2021. The most common problems were wearing the PAM (n=43, 28.7%), reading its display (n=23, 15.3%), logging into its app (n=20, 13.3%), charging it (n=18, 12%), and synchronizing it to the app (n=16, 10.7%). The modalities of technical assistance were in person (n=53, 35.3%), by telephone (n=51, 34%), by email (n=25, 16.7%), and by postal mail (n=21, 14%).

**Conclusions:**

In general, the results of this study show that after receiving orientation to PAMs, problems such as uncomfortable wristbands, difficulty using the PAM or its related app, and obtaining or interpreting relevant personal data were occasionally reported by participants in RS3. Trained staff helped participants troubleshoot and solve these technical problems primarily in person or by phone. Results also underscore the importance of involving older adults in the design, usability testing, and supportive material development processes to prevent technical problems for the initial and ongoing use of PAMs. Clinicians and researchers should further assess technical assistance needed by older adults, accounting for variations in PAM models and wear time, while investigating additional assistance strategies, such as proactive support, short GIF videos, and video calls.

## Introduction

Regular physical activity among older adults, including those with chronic conditions and frailty, confers numerous health and functional benefits [1]. Yet less than 16% of people aged over 65 years in the United States meet the recommendations for minimal physical activity among older adults [2]. Technologies such as commercially available wearable physical activity monitors (PAMs) can prompt some behavior change strategies that help promote regular physical activity [3,4]. Although older adults are interested in using PAMs [5,6], most do not use them [7] or stop using them shortly after obtaining them [8]. An important contributor to their lack of PAM use is the limited information received about their purpose, use, and accuracy at the beginning of ownership and limited structures and resources to provide technical assistance during long-term use [9-14]. Although expert recommendations for providing information at the beginning of PAM ownership are specific, recommendations for providing long-term technical support are general. For example, there is consensus that supportive structures to provide on-demand technical assistance are required to facilitate long-term use [10,15,16], yet specific recommendations are lacking about the type, frequency, and mode of support that might be needed. This paper describes problems reported and technical assistance received by older adults who used PAMs during the 18 months they participated in a community-based physical activity trial (Ready Steady 3.0 [RS3]) [17].

Encouraging older adults to use PAMs can augment efforts to promote increased physical activity, particularly when combined with behavior change strategies such as goal setting, prompts, or social support [1,3,18,19]. Results from recent systematic reviews of physical activity interventions that incorporate PAM use have indicated improved levels of physical activity for at least 6 months across a wide range of age groups, including older adults [20,21]. Additionally, prior research suggests that older adults are interested in and willing to use PAMs [5,6]. Moreover, the *Physical Activity Guidelines for Americans* [1] suggest technology such as PAMs should be used to augment efforts to promote increased physical activity and maintain this increase.

Despite evidence supporting PAM use, there are complex and dynamic problems that limit their adoption and long-term use among older adults. Examples include broken or lost PAMs, perceptions that they are too complicated to learn or use and that their data are inaccurate or not helpful, observations that they are uncomfortable or aesthetically unacceptable, and personal experiences that cause lapses in use (eg, illness) [9,13,22]. As a result, in addition to recommendations for improving several facets of PAM designs, experts have provided specific practical recommendations for improving the technical assistance provided to older adults by clinicians and researchers [15,23]. For example, providing introductory information and instructions (verbal and written, with illustrations) to new PAM owners can boost their understanding about the functions, use, and accuracy of PAMs, as well as how to wear them [16]. After this initial orientation, experts recommend continued assistance through structures such as a technology-help phone line [15]. Planning for such supportive structures raises questions about what to expect regarding the frequency, type, and modalities of such PAM technical assistance.

One strategy to improve our understanding of what is needed to provide long-term structured support for older adults’ use of PAMs is to examine process data regarding their use in a physical activity trial: RS3. The RS3 study aimed to assess the relative effects of 2 intervention components using distinct behavior change techniques on community-dwelling older adults’ physical activity [17]. As part of RS3, participants received and used PAMs (Fitbit Charge 2; Fitbit, Inc) for 18 months [17]. Participants received PAM orientation consistent with published recommendations; they were then encouraged to report PAM problems to the research team and received as-needed technical assistance.

The purpose of this study was to examine the assistance provided to facilitate long-term PAM use among a subsample of RS3 participants. Research questions were as follows:

1. How many participants, after receipt of PAM orientation, reported PAM problems and requested technical assistance during the 18 month follow-up?
  - How many problems were reported or requests made by each participant?
  - What was the rate of unique technical assistance contacts per person-year?
2. What types of problems did participants report that required technical assistance?
3. What modes of technical assistance resulted in resolution of PAM problems?

The results provide a detailed description of the types and quantity of assistance to expect when planning supportive structures to facilitate the long-term use of PAMs among older adults.

## Methods

### Overview

This was an ad hoc, descriptive study using longitudinal process data from 113 of the 309 RS3 participants (Clinical Trials.gov NCT03326141). RS3 was a randomized factorial trial designed to assess the effects of 2 types of behavior change strategies, interpersonal and intrapersonal, in an intervention targeting older adults’ physical activity. The full RS3 protocol is described in another paper [17]. We selected 113 consecutive RS3 participants enrolled between February 2018 and July 2021 as the subsample for this ad hoc study. The reasons for not including the remaining 196 RS3 participants were that (1) they enrolled between November 2017 and February 2018, before ad hoc data collection protocols and procedures were fully operational and systematically captured the types and quantity of PAM technical assistance requested and provided (n=152), or (2) they had yet to complete RS3 (n=44).

### Ethical Considerations

The University of Minnesota’s Institutional Review Board approved and monitored the RS3 trial (1607S90922).

### Participants

Community-dwelling adults enrolled in RS3 and included in this ad hoc substudy were aged ≥70 years, had the ability to walk, had at least 1 fall risk factor [24], had levels of physical activity below national and international recommended guidelines [25], had not had lower extremity surgery or injury in the past 6 weeks, had no diagnosis of neurocognitive dysfunction, and did not have a low score (≤4) on the Callahan Cognitive Screener [26]. Participants received a PAM; an 8-week, small-group intervention; and assessments at 4 time points (baseline, immediately postintervention, 6 months postintervention, and 12 months postintervention). Participants were required to use their PAMs for approximately 2 weeks during each assessment time point and encouraged (but not required) to use their PAMs on a daily basis throughout the study [17]. On average, each participant was involved with RS3 for 18 months and was encouraged to keep and use their PAM after study completion.

### Settings

Technical assistance was provided to participants in community settings, such as community centers, churches, and public libraries, where the RS3 interventions and assessments occurred in Minneapolis and Saint Paul, Minnesota. We also provided assistance in or near participants’ homes, according to participant preferences and COVID-19 restrictions. In addition to community settings, technical assistance was also provided via telephone, postal mail, or email.

### Procedures

#### Training

Part-time research staff were scheduled to cover study implementation, including technical assistance, 9 AM to 5 PM, Monday through Friday. To accomplish this, 12 undergraduate research assistants, 4 graduate research assistants, 3 research professionals, and 3 interventionists were trained to maintain skills and abilities that included facilitating participants’ use of PAMs and their apps, in addition to attaining competencies for their primary roles of assessment, coordination, or intervention delivery. In particular, they all acquired competencies to use PAMs through basic training that involved wearing and using all functions of the PAM in RS3 and downloading and using its app on different platforms (ie, smartphone or computer). Research staff also acquired competencies to effectively communicate with research participants who had previous experiences and varied expertise with PAMs. Staff also learned to use teach-back methods during orientation and technical assistance contacts. Finally, staff acquired coordination and documentation competencies to facilitate the team’s ability to provide timely, successful assistance and track assistance variables.

#### Technical Assistance

Participants received 3 types of technical assistance with their PAMs during RS3: orientation, as-needed technical assistance, and pre- or postintervention meetings (Textbox 1). The timing, strategies and materials used for technical assistance were informed by suggestions and feedback from study participants in RS3 and prior research [27,28].

Textbox 1. **Technical assistance during the Ready Steady 3.0 study.**

**Orientation (one-to-one visits, using checklists for staff and illustrated written instructions for participants)**

- Visit 1
  1. Distribute PAM (physical activity monitor) with charge cable and block.
  2. Describe the purpose and accuracy of the PAM and how to wear and use it.
  3. Explain how to read the PAM display and charge the PAM using the teach-back method.
  4. Provide contact information and encourage participants to call or write research staff with questions before the second assessment.
  5. Conduct a question and answer session.

- Visit 2 (approximately 1 week after the first assessment visit)
  1. Explore participants’ experience with the PAM over the last week and answer questions.
  2. Introduce the PAM app per participants’ access and preferences, help participants download it on their phones, and teach them how to synchronize it, using the teach-back method.
  3. Update the PAM display to include steps, distance, active minutes, and battery level.
  4. Provide information about additional functions and provide assistance programming those functions per the participants’ preferences (eg, reading sleep data).
  5. Encourage participants to contact the researchers with questions and PAM issues and review the phone number for technical assistance.

**As-needed assistance**

1. Monitor study telephone line and emails daily for participants’ technical assistance requests.
2. Respond to participants’ calls and emails immediately or as soon as possible (within 1 to 2 days) using their preferred communication mode.
3. Provide technical assistance over the telephone, by email, and, if necessary, in person.

Orientation to the PAM was standardized and delivered over 2 one-to-one meetings that were guided by the protocol described in Textbox 1, and both were augmented with illustrated guides and the teach-back method [29]. During the first meeting, participants received their PAM and instructions about its purpose, how it works, wearing it, and charging it, with time for questions. During the second meeting, approximately one week after the first meeting, participants were encouraged to ask questions and were given additional information about the PAM display, downloading and using the PAM app, and receiving as-needed technical assistance from the RS3 research team. All orientation meetings were located at the community site of the RS3 study or, when preferred, in participants’ homes.

After the initial orientation, participants were encouraged to contact the research team for any PAM-related questions or issues they experienced through a dedicated study telephone number or email. Research staff were responsible for monitoring the telephone and email. They responded to reports of PAM problems within 1 day or up to 2 days if the request was received on a weekend or holiday. If technical assistance over the telephone, via email, or via postal mail did not solve the reported problem, research staff conducted an in-person meeting based on the participant’s time and location preferences.

During study intervention meetings, participants were encouraged to regularly use their PAMs. The intervention workbooks included a chapter with information about using the PAM, its displays and functions, and how to get as-needed technical assistance help from research staff. However, assistance was not provided during intervention meetings to avoid interfering with the intervention. Instead, when participants requested technical assistance for PAM problems during an intervention meeting, a separate troubleshooting meeting took place after the intervention meeting or was scheduled for another day.

#### Data Collection

Real-time data were collected by trained research staff using computers or tablets and REDCap (Research Electronic Data Capture; Vanderbilt University), where it was verified and managed [30]. The REDCap case report forms included a baseline characteristic questionnaire and a repeatable PAM programming and problem-solving tracker to capture information about technical assistance prospectively over the course of 18 months.

### Variables

Variables to describe sample characteristics included age at study enrollment, sex (female or male), race (Black/African American, Native American, or White), ethnicity (Hispanic or non-Hispanic), and previous experience with the internet (yes or no), smartphones (yes or no), and tablets (yes or no). Although wear time was measured in the main RS3 study during assessment periods, it was not monitored or measured between those periods due to resource limitations. PAM problems and technical assistance processes included dates of contact and assistance, the problem(s) reported (wearing the PAM, charging the PAM, reading the PAM display, logging into the PAM app, syncing the PAM with the app, downloading the app, using a new function on the app, sensor accuracy, sleep data, or a combination of these), and text notes to describe the problem further if needed.

Modes of assistance provided to resolve PAM problems included documentation that interactions were via telephone, email, in-person meetings (eg, in a participant’s home or nearby community center), or postal mail.

Staff also documented when a participant needed a new PAM band (yes or no) and why (broken, uncomfortable, wrong size, or other). Similarly, staff documented when a new device was needed (yes or no) and why (lost, destroyed, malfunctioning, or other).

### Statistical Analysis

Deidentified data from aggregated case report forms were exported from REDCap into SPSS Statistics (version 28.0; IBM Corp) and analyzed using descriptive summary statistics, including means, SDs, and frequencies. Additionally, the incidence rate was calculated as the number of new and distinct participant-research staff contacts for technology assistance per 100 persons per year. Several participants made more than one distinct request during their participation in the study, so each of these distinct contacts was considered a new event and included in this analysis. When new distinct requests involved more than one PAM problem (n=15), the primary/initial problem was used in the frequency and incidence rate calculations.

## Results

### Patient Characteristics

Baseline characteristics of all participants are summarized in Table 1. Data from 113 participants are included in this analysis. A total of 110 participants (97.3%) completed all 18 months of the study. Three participants (2.7%) withdrew due to illness, death, or loss of interest, limiting their follow-up times to 2, 5, and 8 months after enrollment, respectively. On average, participants were aged 76.9 (SD 5.2) years; 87 participants (77%) identified as female, and 92 (81.4%) were experienced smartphone users. As shown in Table 1, participant characteristics were similar across the subsample included in this study and the remaining RS3 sample.

**Table 1.** Characteristics of participants.

Variable		Participants who received PAM^a^ technical assistance	
		Yes (n=113)	No (n=196)
Age (years), mean (SD)		76.9 (5.2)	77.6 (4.9)
Female, n (%)		87 (77)	153 (78)
**Race, n (%)**			
	African American/Black	12 (10.6)	36 (18.4)
	Native American	1 (0.9)	0 (0)
	White	100 (88.5)	155 (79)
	Other	0 (0)	5 (2.6)
**Ethnicity, n (%)**			
	Hispanic/Latino	3 (2.7)	4 (2)
	Non-Hispanic/Latino	100 (88.5)	192 (98)
Living alone, n (%)		51 (45.1)	89 (45.4)
**Self-rated health, n (%)**			
	Excellent	12 (10.6)	18 (9.2)
	Very good or good	87 (77)	159 (81)
	Fair or poor	14 (12.4)	19 (9.8)
**Experience with technology, n (%)**			
	Has used internet	106 (93.8)	168 (85.7)
	Has used a smartphone	92 (81.4)	152 (77.6)
	Has used a tablet	54 (47.8)	80 (40.8)

a PAM: physical activity monitor.

### Participant-Reported PAM Problems Requiring Technical Assistance

Eighty-two participants (72.6%) made between 1 and 9 new and distinct requests for help (150 total) with a total of 169 PAM problems for which they received technical assistance from research staff. Of the 82 participants who made requests, 13 (16%) made 1 request, while 44 (54%), 15 (18%), and 10 (12%) participants made 2, 3, and ≥4 new and distinct requests, respectively. Of the 150 new and distinct requests, 135 (90%) were for 1 PAM-related problem, 11 (7.3%) were for 2 problems, and 4 (2.7%) were for 3 problems. On average, participants in this study made 1.3 (SD 1.3) requests for help with PAM problems. The incidence rate of new, distinct contacts for technical assistance was 99 per 100 persons per year from 2018 to 2021.

**Figure 1.**
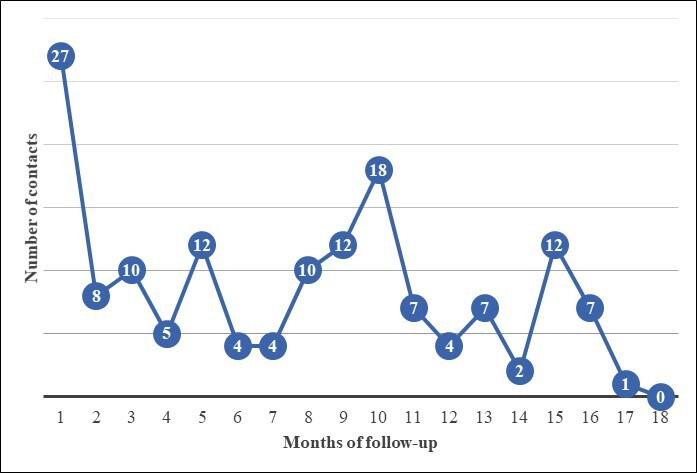
Monthly frequency of physical activity monitor (PAM) problems reported by 82 of 113 study participants requiring technical assistance over 18 months of follow-up after PAM orientation.

Figure 1 illustrates the variation in technical assistance contacts across the 18-month participation time frame. As shown, the frequency of contacts (problems reported and technical assistance provided) peaked during months 1, 10, and 15. These increases corresponded to the study time frames just after initial PAM orientation and the 6- and 12-month research assessments.

### Types of PAM Problems and Modes of Technical Assistance

**Figure 2.**
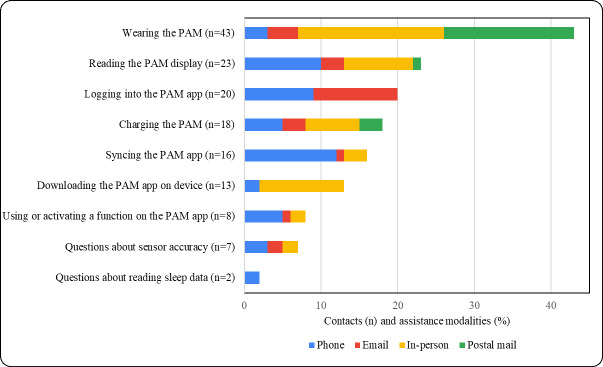
Physical activity monitor (PAM) problems and types and modes of technical assistance.

Types, frequencies, and modes of requests for help with PAM problems are illustrated in Figure 2. Among the 150 requests, the most common problem was with (1) wearing the PAM (n=43, 28.7%), followed by (2) reading the PAM display (n=23, 15.3%), (3) logging into the PAM app (n=20, 13.3%), (4) charging the PAM (n=18, 12%), (5) synchronizing the PAM app (n=16, 10.7%), (6) downloading the PAM app to a device (n=13, 8.7%), (7) using or activating a function on the PAM app (n=8, 4.7%), (8) having questions about sensor accuracy (n=7, 4.7%), and (9) reading sleep data (n=2, 1.3%). The modalities used in the 150 contacts to provide solutions for technical assistance included 53 (35.3%) in-person visits at community centers, participants’ homes, or locations of their preference in their neighborhoods (eg, a church, library, or coffee shop); 51 (34%) telephone conversations; 25 (16.7%) email conversations; and 21 (14%) notes and instructions sent via postal mail. Twenty-seven of the 150 contacts (18%) comprised multiple visits and modes because initial technical assistance via telephone did not resolve the problem; this was particularly the case for programming or equipment problems.

In addition to technical assistance, 35 of the 150 (23.3%) PAM problems reported required research staff to distribute new bands, and 5 (3.3%) required new PAM devices. New bands were provided to improve comfort (n=5), improve the ease of fastening the Fitbit (n=16), or improve preferred aesthetics (n=14), such as by using a different color or material. New PAMs were provided due to malfunction (n=2) or loss (n=3).

## Discussion

### Principal Findings

In this study, we examined the incidence and type of distinct PAM problems reported by older adults participating in an 18-month research trial that required technical assistance to resolve. We also analyzed the mode of technical assistance provided in this study. Our findings suggest that after an initial orientation to PAMs, researchers, clinicians, and others providing technical assistance to facilitate long-term use can expect at least 99 technical assistance contacts per 100 persons per year. Variations across the 18-month follow-up time in this study showed upward trends immediately after PAM orientation and when research staff contacted participants to coordinate study assessments. The problems reported were mostly solvable and related to comfort, using the PAM device and app, and interpreting data. Finally, approximately one-third of the technical assistance contacts were in person, and the remainder were via telephone, email, or postal mail.

To our knowledge, the prior literature has not described the frequency or rate of participants’ requests for assistance with PAM technology immediately after receipt or over an extended period. Thus, our findings advance the prior literature, which has elucidated reasons for the limited adoption and use of PAMs [9,13] and recommended improvements to technical assistance provided to older adults who are beginning to use commercially available PAMs, as well as those who use them long term [15,16].

This study supports a new, developing conceptual model [10], which argues that for a person to integrate a PAM into daily life, they initially need help setting it up and learning about its features, and they then need technical assistance over time. It also supports and extends expert recommendations to provide initial orientation and then to maintain supportive structures for as-needed technical assistance. This can facilitate older adults’ long-term use of PAMs [15,31] by providing details about the anticipated types and frequency of ongoing assistance. Implications for this finding are that those who encourage older adults to use PAMs (eg, family, friends, clinicians, researchers, public health professionals, and health promotion programs) should plan for providing occasional technical assistance over time. Furthermore, it would be beneficial to combine responsive and proactive strategies for providing technical assistance, given our observation of more technical assistance contacts during months when research staff reached out to participants. That is, in addition to expecting occasional reports of PAM problems that need assistance, it may be helpful to occasionally initiate contacts with PAM users to invite questions and identify problems.

Our findings about the types of problems observed in this study were consistent with previous research that categorized reasons for abandonment as problems related to the PAM, user preferences, or the PAM app [16,32]. The most common problem observed was wearing the out-of-the-box PAM wristband, whose rubber material and clasp were difficult to use, unappealing to some, or broke, which underscores the importance of helping people customize their wearable devices for comfortable everyday use [15,33]. Problems identified by participants, such as reading the PAM display, logging into the PAM app, and synchronizing the PAM to the app, have been identified in prior literature as common usability issues that prevent access to personal physical activity data [16,34]. Our findings support prior recommendations for future design and optimization of wearable technology for older adults [35-37] and demonstrate that many of the problems we observed were solvable with technical assistance.

Lastly, our finding that multiple modes were used to provide PAM-related technical assistance suggests that flexibility is essential. Many technical assistance contacts via phone, email, or postal mail successfully solved the reported problems. Nevertheless, some PAM problems were solved only after in-person interactions within participants’ homes or nearby community centers, according to their preference. This finding raises questions about whether additional assistance strategies, such as short GIF videos and video calls, might efficiently augment strategies to provide technical assistance, while also reducing the need for some in-person contacts.

### Limitations

This study has several limitations. One is the possibility that we underestimated the frequency and rate of unique technical assistance contacts due to the design of our technical assistance structures in RS3 and our data collection approach. In RS3, we focused on responding to participants’ reported PAM problems and requests for assistance. It is possible that a more proactive approach (eg, occasionally reaching out to participants) would uncover additional PAM problems or gradually mitigate problems over time, thereby changing our longitudinal observations. Our data collection procedures focused on interactions between participants and research staff. It is possible that some participants reported PAM problems or requested technical assistance from other people, such as friends, neighbors, family, clinicians, or Fitbit Inc. A second limitation was that we did not collect data about wearing behavior [38] outside the assessment periods. Thus, we cannot analyze the extent to which technical assistance was associated with longitudinal daily PAM-wearing behaviors, and we recommend future studies investigate this. A third limitation is that the RS3 study included only one model of PAMs. As the design of PAMs changes over time and varies between different makes and models, the frequencies and types of technical assistance required by users may also shift.

### Conclusions

Encouraging older adults to use PAMs can augment efforts to promote increased physical activity, but adequate technical assistance is needed. Findings from this study show that after receiving orientation to PAMs, problems such as uncomfortable wristbands, difficulty using the PAM or its related app, and obtaining or interpreting relevant personal data are reported. With occasional technical assistance, these problems are solvable over the phone, by email, or by postal mail. However, depending on the problem and an older person’s preferences, some PAM problems are best solved in person. The detailed description of PAM problems and contacts provided in this study can inform plans for the quantity, types, and modes of technical assistance required from supportive structures that facilitate the long-term use of commercially available PAMs among older adults. Our results underscore the importance of trying to prevent technical problems by involving older adults in the design of PAMs, from initial design to usability testing and the development of supportive materials and structures for initial and ongoing use. Clinical programs and researchers should further assess the technical assistance needed for PAM problems experienced by older adults, accounting for variations by PAM model and wear time. Future research should also investigate additional assistance strategies, such as proactive support, short GIF videos, and video calls.

## Abbreviations:

PAM: physical activity monitor
REDCap: Research Electronic Data Capture
RS3: Ready Steady 3.0
